# Changes in gene expression patterns in postmortem human myocardial infarction

**DOI:** 10.1007/s00414-020-02311-2

**Published:** 2020-05-12

**Authors:** Verena Wilmes, Constantin Lux, Constanze Niess, Elise Gradhand, Marcel A. Verhoff, Silke Kauferstein

**Affiliations:** 1 Institute of Legal Medicine, Johann Wolfgang Goethe University, Frankfurt, Germany; 2 Institute of Pathology, University Hospital Frankfurt, Frankfurt am Main, Germany

**Keywords:** Inducible nitric oxide synthase (iNOS), Hypoxia-inducible factor-1α (HIF-1α), Vascular endothelial growth factor (VEGF), Myocardial infarction (MI), Hypoxia, Transcription regulation

## Abstract

In murine models, the expression of inducible nitric oxide synthase (iNOS) in myocardial infarction (MI) has been reported to be the result of tissue injury and inflammation. In the present study, mRNA expression of iNOS, hypoxia-inducible factor-1α (HIF-1α), and vascular endothelial growth factor (VEGF) was investigated in postmortem human infarction hearts. Since HIF-1α is the inducible subunit of the transcription factor HIF-1, which regulates transcription of iNOS and VEGF, the interrelation between the three genes was observed, to examine the molecular processes during the emergence of MI. iNOS and VEGF mRNAs were found to be significantly upregulated in the affected regions of MI hearts in comparison to healthy controls. Upregulation of HIF-1α was also present but not significant. Correlation analysis of the three genes indicated a stronger and significant correlation between HIF-1α and iNOS mRNAs than between HIF-1α and VEGF. The results of the study revealed differences in the expression patterns of HIF-1 downstream targets. The stronger transcription of iNOS by HIF-1 in the affected regions of MI hearts may represent a pathological process, since no correlation of iNOS and HIF-1α mRNA was found in non-affected areas of MI hearts. Oxidative stress is considered to cause molecular changes in MI, leading to increased iNOS expression. Therefore, it may also represent a forensic marker for detection of early changes in heart tissue.

**Electronic supplementary material:**

The online version of this article (10.1007/s00414-020-02311-2) contains supplementary material, which is available to authorized users.

## Introduction

Myocardial infarction (MI) is the result of sustained insufficient blood supply to areas of the myocardium [1]. Restoration of the blood flow to the ischemic myocardium causes the generation of reactive oxygen species (ROS) [2], the activation of inflammatory cascades, and the expression of the inducible nitric oxide synthase (iNOS) [1, 3]. Induction of iNOS occurs in response to stimuli like inflammation, tissue injury, and hypoxia [4]. In murine models of infarction [5, 6] and in postmortem human infarction hearts [7], increased iNOS expression has been detected, suggesting that iNOS contributes to MI by its ability to produce large amounts of nitric oxide (NO), which has negative inotrope effects [1] and is cytotoxic at high levels [8]. Furthermore, excessive NO production by iNOS is accompanied by increased ROS production including superoxide and peroxynitrite, which have detrimental effects to the heart [9–11]. iNOS as a producer of ROS is considered to be an indicator of oxidative stress. Several studies suggest that it plays a pathological role in MI and ischemia reperfusion injury [1, 4–6, 12]. Increased iNOS expression may already occur during the development of MI, leading to a poor outcome through the generation of oxidative stress in the myocardium. However, the implication of iNOS in cardiovascular diseases is still controversially discussed, as some studies suggest rather beneficial than harmful effects [9, 10, 13–15].

In forensic approaches, cardiac oxidative stress is considered to contribute to cardiac death, aggravating postmortem diagnosis [16]. Furthermore, in humans who died of acute exposure to high doses of cocaine, myocardial oxidative damage was found through oxidative stress, accompanied by a significant increase in iNOS protein [17]. Thus, iNOS may be a possible forensic marker of myocardial oxidative stress.

Besides the proinflammatory signaling pathway [18, 19], the transcription factor hypoxia-inducible factor-1 (HIF-1) regulates iNOS expression under hypoxic conditions [13, 15]. HIF-1 is critical for initiating early responses to hypoxia and it is the main modulator in adapting gene expression [20–22]. As a transcriptional complex, it consists of the subunits HIF-1β and HIF-1α. While HIF-1β is constitutively expressed, the HIF-1α expression depends on the cellular oxygen level [13]. Under normoxic conditions, proline and asparagine residues in HIF-1α are hydroxylated, leading to a proteosomal degradation or inhibition of binding to co-activator proteins [22]. Under hypoxic conditions, the hydroxylation is suppressed. HIF-1α subunits are translocated into the nucleus and bind with HIF-1β to form the transcription factor HIF-1 [23] which elicits a wide range of adaptive responses, important for tissue protection and adaption [22]. HIF-1 is known to be associated with the upregulation of iNOS under hypoxic conditions [22, 24] and also induces the vascular endothelial growth factor (VEGF), which is important to improve oxygen supply by stimulating revascularization to limit ischemic damage of the heart [22].

Several studies examined the interaction between HIF-1α, VEGF, and iNOS in human and murine MI models. However, they mainly focused upon the expression patterns of HIF-1α and iNOS in ischemia reperfusion injury in murine models [14, 25]. In humans, Marfella et al. [12] investigated the interplay of the three genes in diabetic patients with angina pectoris but not in manifested MI, describing a rather detrimental role of iNOS, due to the combination with oxidative stress. While HIF-1α and VEGF are considered to have beneficial effects in the ischemic heart [26–30], the induction of iNOS is still controversially discussed [31, 32].

In the present study, the expression pattern of HIF-1α versus that of VEGF and iNOS in human infarction hearts was investigated, to further clarify the expression of iNOS in MI and its possible role as a forensic marker for cardiac oxidative stress. Furthermore, HIF-1α is one of the first genes upregulated in hypoxia and its expression may be influenced by factors such as the postmortem interval (PMI) and cause of death.

## Materials and methods

### Ethical approval

No informed consent was required. This work was approved by the local ethics committee.

### Sample selection

Cardiac tissue samples from 22 deceased individuals were collected during autopsies at the Institute of Legal Medicine, Goethe University Frankfurt, Germany. Samples from healthy hearts (*n* = 7, 5 males, 2 females, mean age 28 years, mean PMI 3 days) as control and from hearts with macroscopically visible signs of acute cardiac infarction (MI hearts; *n* = 15, 11 males, 4 females, mean age 67 years, mean PMI 6 days) were histologically examined and RNA was extracted. From the infarction area of the MI hearts, two replicates were taken, either from the posterior or anterior wall, depending on the infarct localization, two from the macroscopically unaffected wall of the MI hearts and two from the anterior and posterior wall of control hearts. Variations within one heart and the heterogeneity of the infarctions were considered by taking more than one tissue sample. All samples were obtained from the left ventricle.

The study and the control groups are shown in Tables 1 and 2.

**Table 1:** Age, sex, PMI, and histology data of the study group. In samples F4–F6, because of the large infarction area, no unaffected tissue could be taken

Sample	Age	Sex	Histology: affected area	Histology: unaffected area	PMI (day)
F1	66	Male	Acute infarction and older infarction area	No signs of cell damage	4
F2	75	Female	Acute, diffuse infarction	No signs of cell damage	6
F3	70	Male	Acute, diffuse infarction	Very small scar, i.e., a sign of previous ischemic event or myocarditis	7
F4	76	Female	Acute, diffuse infarction	-	5
F5	58	Male	Scar tissue	-	1
F6	88	Female	Diffuse infarction	-	8
F7	70	Male	Scar tissue	No signs of cell damage	9
F8	70	Male	Scar tissue	No signs of cell damage	12
F9	59	Male	Acute infarction	Slight fibrosis	5
F10	45	Male	Diffuse, subacute infarction	Focal, subacute cell damage	4
F11	69	Female	Acute infarction	Acute hypoxia	3
F12	76	Male	Diffuse, older infarction	Fibrosis	9
F13	44	Male	Overlapping of acute infarction and older infarction area	No signs of cell damage but acute hypoxia	7
F14	54	Male	Acute, focal infarction	No signs of cell damage	8
F15	91	Male	Acute infarction, macular fibrosis	No signs of cell damage	6

**Table 2:** Age, sex, PMI, and cause of death of the control group

Sample	Age	Sex	Cause of death	PMI (day)
K1	2	Male	Suffocation	3
K2	36	Male	Suicide, death by hanging	1
K3	18	Male	Suicide, hit by train	6
K4	47	Male	Traffic accident	4
K5	24	Male	Suicide, jumping from high building	4
K6	21	Female	Drowning	5
K7	49	Female	Suicide, jumping from high building	1

### Histological examination

Tissue samples were fixed in 4.5% buffered formalin and embedded in paraffin, and sections of 5 μm were stained with hematoxylin-eosin.

### Reverse transcription quantitative polymerase chain reaction

One hundred milligrams of cardiac tissue was, by using a pestle, mechanically homogenized in 1 mL Trizol (TRI-Reagent, Sigma-Aldrich, Taufkirchen, Germany) and incubated for 30 min at room temperature. Samples were stored at − 80 °C until RNA isolation or were processed immediately. RNA was isolated according to the manufacturer’s protocol. Genomic DNA was digested using the TURBO DNA-free™ Kit, following the manufacturer’s protocol (Thermo Fisher Scientific, Schwerte, Germany). Success of total DNA digestion was verified by amplification of a region of the mitochondrial cytochrome oxidase subunit 1. The primers for this region are established in different forensic approaches, especially verification of DNA digestion, at the Institute of Legal Medicine in Frankfurt. Afterwards, the extracted RNA samples were purified according to the protocol of the RNeasy Mini Kit (Qiagen, Hilden, Germany). The RNA concentration was measured using Qubit® 3.0 Fluorometer and Qubit® RNA BR Assay Kit (Life Technologies GmbH, Darmstadt, Germany). For synthesizing single-stranded cDNA from extracted RNA templates, the High-Capacity cDNA Reverse Transcription Kit (Applied Biosystems, Darmstadt, Germany) was used according to the manufacturer’s protocol. The priming conditions were random. The thermocycler program is described in Table 3.

**Table 3:** Thermocycler program for cDNA synthesis

Step	Temperature (°C)	Duration (h)
1	25	00:10
2	37	02:00
3	85	00:05
4	4	Stop

Quantitative real-time PCR was performed using the following Taq Man® Gene Expression Assays: inducible nitric oxide synthase (NOS2, amplicon size 67 bp, assay ID: Hs01075529_m1, refseq.-nr.: NM_000625.4), hypoxia-inducible factor-1α (HIF1A, amplicon size 76 bp, assay ID: Hs00153153_m1, refseq.-nr.: NM_001530.3), vascular endothelial growth factor (VEGF, amplicon size 59 bp, assay ID: Hs00900055_m1, refseq.-nr.: NM_003376.5). Peptidylprolyl isomerase A (PPIA, amplicon size 98 bp, assay ID: Hs99999904_m1, refseq.-nr.: NM_021130.4), TATA box–binding protein (TBP, amplicon size 91 bp, assay ID: Hs00427620_m1, refseq.-nr.: NM_003194.4), and tumor protein translationally controlled 1 (TPT1, amplicon size 131 bp, assay ID: Hs02621289_g1, refseq.-nr.: NM_003295.3) (Applied Biosystems, Darmstadt, Germany) were used as endogenous reference genes due to their postmortem stability and their stable expression in cardiac tissue [33, 34]. For each sample, triplicates were applied as well as negative controls for each assay. Negative controls included no-RT controls and PCR-negative controls. The qPCR reaction mix and the cycler program are described in Tables 4 and 5.

**Table 4:** Reaction mix in the qPCR per each well

Reagent	Volume (μL)
Maxima probe/ROX qPCR Master Mix	5
TaqMan-Gene Expression Assay	0.5
cDNA	1–4.5 (200 ng)
Nuclease-free water	Ad 10

**Table 5:** Program of the qPCR

Step	Temperature (°C)	Duration (min)
1	50	02:00
2	95	10:00
3	85	00:15
4	60	01:00
5	Go to 3	40×

Analyzing the qPCR efficiency was performed by using LinReg PCR software [35]. The program determines a baseline fluorescence and a window of linearity is set. The linear regression analysis of the gradient was used to determine PCR efficacy per sample. The qPCR was efficient when the values were between 1.8 and 2.0. Values lower than 1.8 indicate an inadequate efficacy and were excluded. Efficacy values per heart assay were determined and used to correct the corresponding Cq values.

For qPCR of iNOS, 200 ng of HIF-1α and VEGF and 100 ng cDNA were used. After reverse transcription, cDNA concentrations were not measured, since the reverse transcription ratio of RNA:cDNA is 1:1.

The differences in the input amounts stem from limited sample amounts.

### RNA integrity number measurements

For evaluation of the RNA degradation in cardiac tissue samples, the RNA integrity number (RIN) was determined. Sample preparation was performed according to the manufacturer’s protocol (RNA ScreenTape System, Agilent Technologies, Ratingen, Germany) and samples were analyzed using the Agilent 4200 TapeStation (Agilent Technologies, Ratingen, Germany). A RIN value of 10 and a 28S/18S ratio of > 2 indicate that no RNA degradation had occurred, whereas a value of 1 and no measurable 28S/18S ratio indicate highly degraded RNA.

### Data analysis

qPCR was carried out by using the Software 7500, version 2.0.6, and Data Assist v3.01 served for analyzing the qPCR raw data. Calculation of fold change was carried out using the ΔΔCq method [36]:

- ΔCq = average Cq of the triplicates of the gene of interest − normalization factor of the endogenous references
- ΔΔCq = ΔCq of the gene of interest − ΔCq of the calibrator sample
- 2^-ΔΔCq = 2^-(ΔCq of gene of interest − ΔCq of the calibrator sample)

The software R version 3.5.1 was used for statistical analysis. Linear mixed effects model and pairwise post hoc comparisons were applied and significance was adjusted by using Tukey’s multiple comparisons test. Two individual measurements of the unaffected and the affected regions of MI hearts were included in the statistical analysis to detect local differences in the gene expression per heart and two of the anterior and posterior wall for each control heart. Furthermore, after the Mudholkar test of bivariate Gauß distribution, Pearson and Spearman correlations were applied. Pearson correlations were applied, when the test of Gauß distribution revealed a normal distribution. In other cases, Spearman correlations were applied.

Receiver operating characteristic (ROC) analysis was applied for evaluating the diagnostic power of iNOS. The area under the curve (AUC) was calculated. Considering dependencies, due to multiple samples of the same heart, a general linear mixed effects forecast model was applied.

### geNorm

The stability of the reference genes in the postmortem samples was evaluated by geNorm [37]. Koppelkamm et al. [33] investigated the chosen reference genes PPIA and TBP using geNorm already in a set of postmortem cardiac tissue samples, and described them as most stable. TPT1 is described as stable expressed in cardiac tissue [34]. Therefore, we performed the geNorm analysis to examine the *M* value in the given set of samples for these genes.

## Results

### Sample selection

Macroscopic pathological findings were confirmed by histological examination (Table 1), revealing subacute, acute, or old infarction areas, either focal or diffuse. The non-infarcted areas showed no cell changes or cardiomyocyte injuries, except in one case (F10), indicating focal subacute injury due to the presence of single neutrophils (Table 2). The control group samples did not exhibit pathological cardiac changes.

### RNA integrity number measurements

A mean RIN of 1.8 (± 0.7) was measured in the affected infarction areas and of 1.9 (± 0.8) in the non-affected areas of MI hearts. In healthy control hearts, a mean RIN of 3.5 (± 1.3) was detected. The RIN values measured in the affected as well as non-affected areas of MI hearts indicate RNA degradation. Although the RNA quality in the control group is increased, the RIN values still imply degraded RNA.

### Stability of endogenous reference genes

The stability of the genes was determined by stability measures *M*. Measured *M* values obtained by geNorm revealed medium postmortem stability of the endogenous reference genes: 0.749 (TPT1), 0.743 (TBP), and 0.721 (PPIA) for HIF-1α; 0.696 (TPT1), 0.680 (TBP), and 0.654 (PPIA) for VEGF; and 1.040 (TPT1), 0.960 (TBP), and 0.897 (PPIA) for iNOS.

### ROC analysis

**Fig. 1:**
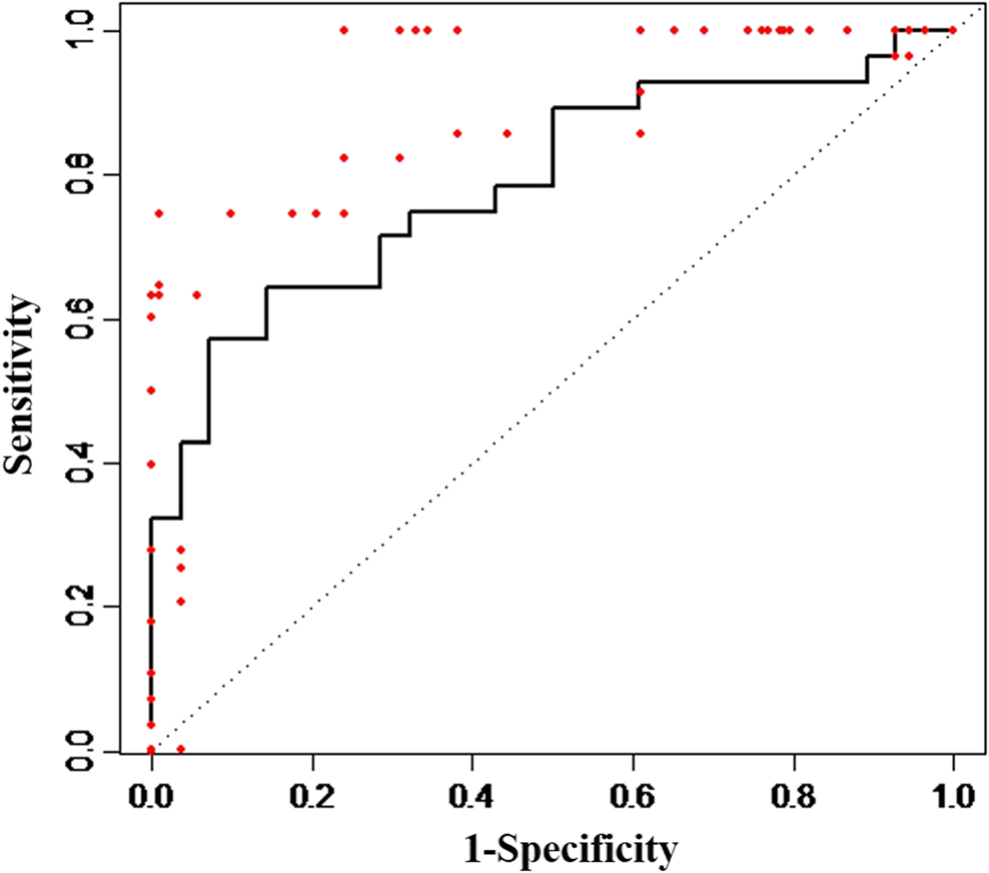
ROC curve analysis. The black line demonstrates the ROC curve, neglecting the dependencies in the samples. AUC = 0.78. The red dots imply the shift in the ROC curve, when the dependencies are regarded

For estimation of the diagnostic accuracy of iNOS, a ROC curve analysis was performed (Fig. 1). When neglecting the dependencies, the AUC equals 0.78. Therefore, a higher iNOS value is measured in the infarction group, as in 78% of the healthy controls. At a threshold value of 0.74 (-ΔΔCq), the sensitivity equals 0.642 and the specificity 0.857. When considering the dependencies, it was not possible in R to draw a ROC curve, with a subsequent AUC. However, the red dots in Fig. 1 display the curve when the dependencies of multiple samples of the same heart are considered. As can be seen, the ROC curve shifts, increasing the diagnostic accuracy of iNOS. Thus, the sensitivity at the threshold value of 0.74 (-ΔΔCq) equals 0.745 and the specificity 0.99.

### Increased mRNA expression in MI hearts

**Fig. 2:**
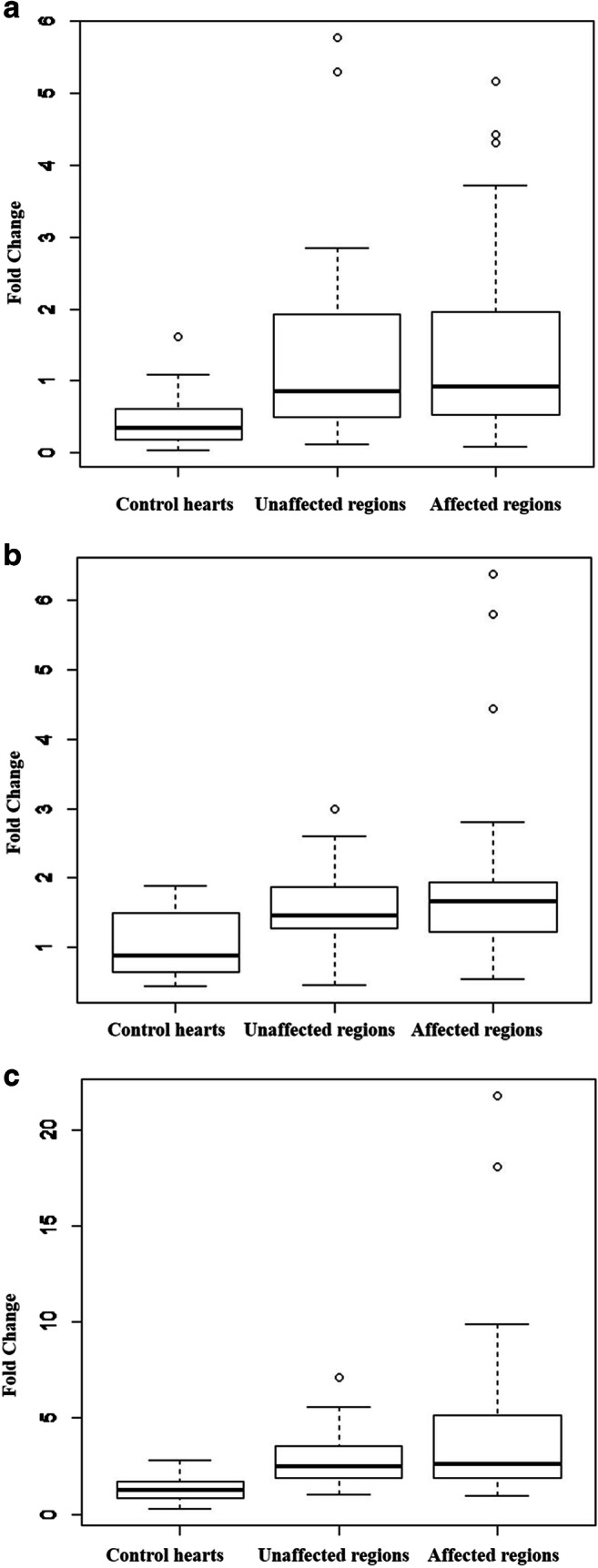
**a** iNOS expression in control hearts (*n* = 7), in unaffected (*n* = 12) and in affected (*n* = 15) regions of MI hearts. The expression is clearly upregulated. Some samples of the unaffected areas show the same expression pattern as in the affected regions. **b** HIF-1α expression in control hearts (*n* = 5) and in unaffected (*n* = 10) and in affected (*n* = 13) regions of MI hearts. The expression is increased in MI hearts; however, in some controls, the expression of HIF-1α is increased as well. **c** VEGF expression in control hearts (*n* = 4) and in unaffected (*n* = 9) and in affected (*n* = 11) regions of MI hearts. The VEGF expression is strongly increased compared to control hearts. In all gene expression experiments, sample K4 was set as the calibrator sample

iNOS expression in control hearts and in both non-affected and affected regions of MI hearts is shown in Fig. 2a. In the affected regions, the iNOS expression is significantly increased (*p* = 0.03) in comparison to the healthy control hearts. The non-affected regions of MI hearts show a tendency towards some, but not significantly increased iNOS expression (*p* = 0.13). In individual samples, however, the non-affected regions exhibit the same and even higher iNOS levels than the affected regions.

A strong but not significant upregulation of HIF-1α was observed in the affected regions (*p* = 0.09) and a slight upregulation in the non-affected regions of MI hearts in comparison to healthy control hearts (Fig. 2b).

In the affected regions of MI hearts, a significant increase (*p* = 0.03) of VEGF mRNA in comparison to healthy control hearts was found, but upregulation of VEGF mRNA in the non-affected regions of MI hearts was not significant (*p* = 0.08) although there is a tendency towards increased VEGF mRNA expression in these regions when compared to the healthy controls.

In each described mRNA expression experiment, sample K4 was set as the calibrator sample, since with age and gender it matched best to the majority of the study group. Thereby, K4 was also used to calibrate the control group.

### Correlation between HIF-1a, VEGF, and iNOS mRNA expression

**Fig. 3:**
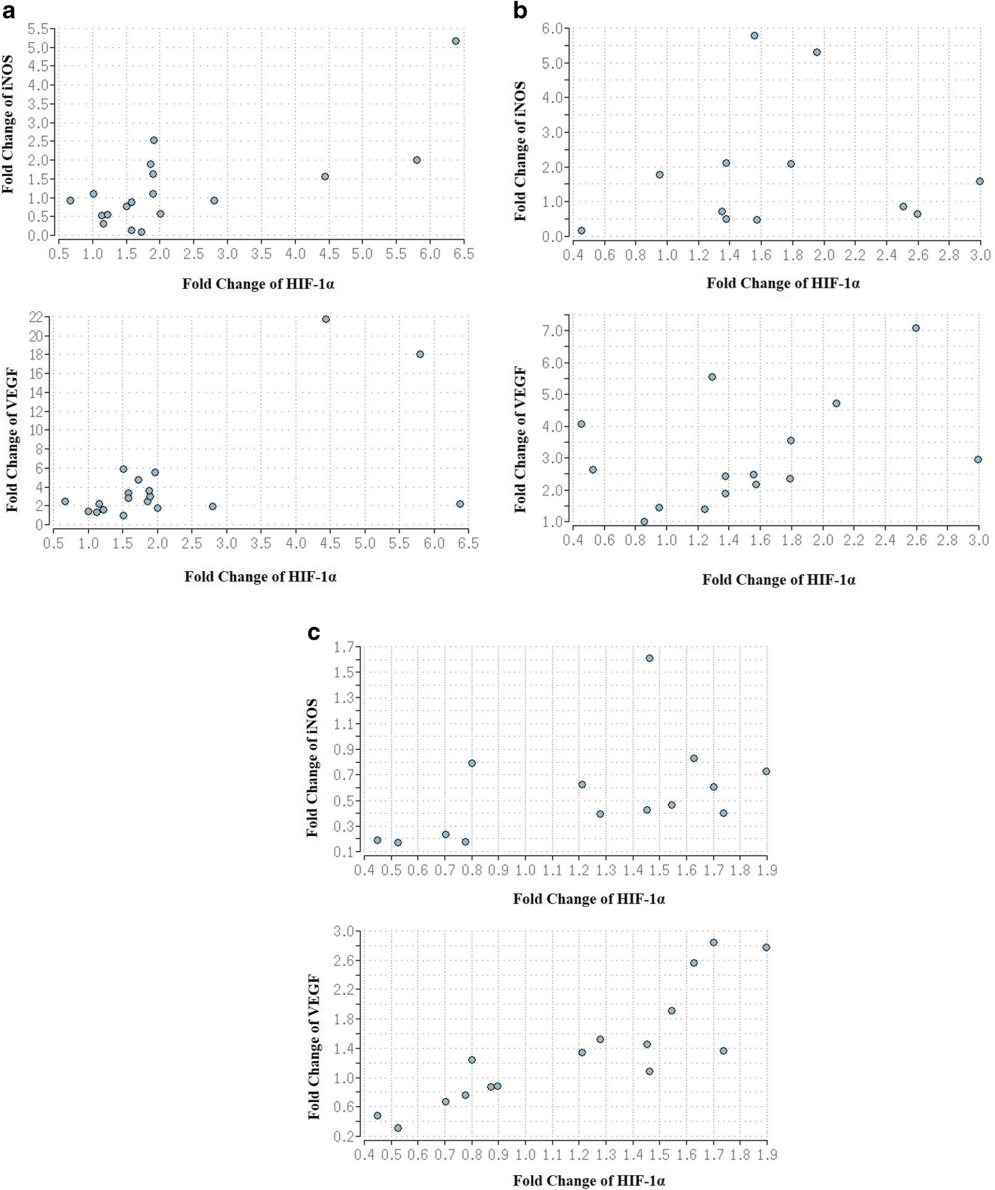
**a** Scatterplot of the monotone relation between HIF-1α and iNOS mRNA in affected regions of MI hearts (top). Scatterplot of the monotone relation between HIF-1α and VEGF mRNA in affected regions of MI hearts (bottom). The relation between HIF-1α and iNOS is stronger than between HIF-1α and VEGF as indicated by the dots. **b** Scatterplot of the monotone relation between HIF-1α and iNOS mRNA in unaffected regions of MI hearts (top). Scatterplot of the monotone relation between HIF-1α and VEGF mRNA in unaffected regions of MI hearts (bottom). Here, the relation between HIF-1α and VEGF is more prominent than between HIF-1α and iNOS. **c** Scatterplot of the monotone relation between HIF-1α and iNOS mRNA in control hearts (top). Scatterplot of the monotone relation between HIF-1α and VEGF mRNA in control hearts (bottom)

For analysis of the interrelation between these three genes in MI hearts, Spearman’s correlation was applied, which revealed a moderate (rho = 0.53) and significant (*p* < 0.05) monotone relation between HIF-1α and iNOS mRNA expression in the affected regions of MI hearts. Furthermore, a less moderate (rho = 0.45) and not significant (*p* < 0.1) monotone relation was detected between HIF-1α and VEGF mRNA expression in the affected regions of MI hearts (Fig. 3a).

In the unaffected regions of MI hearts, no monotone relation (rho = 0.22) between HIF-1α and iNOS was found, but there was a moderate monotone relation (rho = 0.4) between HIF-1α and VEGF, which was not significant (*p* < 0.2) (Fig. 3b).

In control hearts, the monotone relations between HIF-1α and iNOS (rho = 0.6) and HIF-1α and VEGF (rho = 0.89) gene expression were significant (*p* < 0.05) (Fig. 3c).

### Correlation between RIN/PMI and HIF-1α, VEGF, and iNOS mRNA expression

To analyze whether the expression of iNOS, HIF-1α, and VEGF was influenced by the PMI or RIN, Pearson correlations were applied and scatterplots were used for data presentation. No linear correlation between PMI/RIN and the expression of the three genes in MI hearts as well as in control hearts was detectable (data not shown).

## Discussion

In the present study, iNOS and VEGF gene expression in the affected regions of MI hearts was found to be significantly increased when compared to healthy control hearts, while HIF-1α expression was increased as well, but not significantly. In the non-affected areas of MI hearts, iNOS, VEGF, and HIF-1α mRNA were found to be upregulated.

In rat models, Li et al. [6] and Takimoto et al. [38] observed increased iNOS expression in MI and attributed it to inflammatory reactions after infarction. However, in the present study, iNOS was not upregulated due to infiltration of neutrophils in the acute inflammatory phase following MI, because histological examination revealed no tissue injuries (except for F10) in the non-affected areas. The tendency to an increased iNOS expression in the non-affected myocardium supports the assumption that iNOS is involved early in the development of MI. Oxidative stress as a result of hypoxia or cardiac overload may already occur before the complete occlusion of the arteries, inducing the expression of iNOS before signs of infarction can be detected.

On the other hand, Takimoto et al. [38] suggested that cytokines promote iNOS expression in cardiomyocytes of non-affected regions after MI, since cytokine levels are elevated after infarction.

In the study of the oxidative stress pathway in MI hearts, HIF-1α, the inducible subunit of a transcription factor of iNOS, showed a strong upregulation of its mRNA in the affected regions as demonstrated in previous studies [12, 30]. Furthermore, the increase of HIF-1α mRNA expression in the non-affected regions of the heart is assumed to be a sign of early myocardial hypoxia.

These results are in contradiction to findings of Lee et al. [30] and Marfella et al. [12]. They found that in the cases of human myocardial infarction and of unstable angina pectoris, HIF-1α mRNA expression was strictly confined to the ischemic area, but was not detectable in non-ischemic parts of MI hearts. Lee et al. [30] concluded that HIF-1α mRNA expression is initiated very early after the onset of myocardial ischemia or infarction to control the expression of genes inducing angiogenesis. One possible explanation for the contradictory results is different analytical methods applied. While Marfella et al. [12] and Lee et al. [30] used reverse transcriptase PCR and quantification based on visualized gel electrophoresis bands, in the present study, qPCR was performed, which is a more sensitive approach to detect mRNA levels of HIF-1α and may explain its detection in the non-affected areas.

This may indicate that even before cell injuries can be detected, hypoxia is already present, subsequently activating downstream genes like iNOS and leading to oxidative stress in MI.

Control and study groups differ in their mean age. However, no influence on the gene expression has been observed. When comparing the expression levels of samples of the same or very similar age (K4, K7, F10, F13, and F14), no expression pattern of iNOS, HIF-1α, or VEGF was found. While the tissue samples of F13 showed no iNOS mRNA upregulation in the affected areas, the samples of F10 and F14 showed the strongest iNOS increase in the whole study group. Furthermore, to the best of our knowledge, no influence of age to the iNOS mRNA expression has been reported.

The lack of a correlation between HIF-1α mRNA expression and the postmortem interval (PMI) is in contrast to the study of Fais et al. [39]. In the cases of PMIs between 1 and 5 days, they reported an increase in HIF-1α mRNA expression in gingival tissue, but when the PMI was longer than 5 days, the HIF-1α expression decreased and was no longer detectable at a PMI of 10 days. Vennemann and Koppelkamm [40] stated that the endogenous reference gene GAPDH, which was used by Fais et al. [39], is not a stable reference gene, because it is influenced by hypoxia. The application of three stable reference genes may explain the different results of the present study, which are in accordance with studies of Zhu et al. [41] and Zhao et al. [42], indicating that HIF-1α is a good marker of hypoxia, because its expression is not influenced by gender, age, and PMI.

HIF-1 is also the transcription factor of VEGF, an inducible factor responsible for capillary growth and angiogenesis in various organs [30]. In chronically ischemic myocardium and after MI in rats, VEGF mRNA was significantly increased due to hypoxia [43] and its expression in the myocardium is seen in macrophages and myocytes [29, 43]. Li et al. [43] investigated the distribution of VEGF expression after myocardial infarction in rat hearts and observed increased VEGF mRNA expression in infarcted and non-affected myocardium of the left ventricle. The increase in the myocardium remote from the infarction area was significant and higher than the increase in the infarcted area. They concluded that the expression of VEGF in the periphery of the affected myocardium indicates the beginning of angiogenesis at the border of the infarct area. Lack of oxygen supply in the non-ischemic areas of MI hearts may also contribute to the increase of VEGF expression [43]. The results of the present study support these results, as upregulation of VEGF in the non-affected myocardium was also observed. On the other hand, Shinohara et al. [29] reported only weak VEGF expression in affected and non-affected regions of human MI hearts, which may be explained by the different, probably less sensitive method used.

### Interaction between HIF-1α, iNOS, and VEGF in myocardial infarction hearts

The relation of these three genes in different ischemic heart models has been investigated by several authors [12, 22, 31]. Natarajan et al. [25] described a protective effect of HIF-1α overexpression and subsequent enhanced iNOS activation in a murine model of ischemia reperfusion injury. Furthermore, beneficial effects on the myocardium by HIF-1α and iNOS were reported in ischemic preconditioning before MI and subsequent reperfusion treatment [15, 13]. However, during reperfusion treatment, iNOS causes a large burst of NO, as well as of superoxidanion, leading to the formation of peroxynitrite, hence oxidative stress [44]. Thereby, reperfusion is thought to cause additional myocardial damage [45]. Under physiological conditions, there is an equilibrium between formation and clearance of ROS, which is mostly based on antioxidant enzymes. In the heart, the most important antioxidative enzymes are superoxide dismutase (SOD), catalase (CAT), and glutathione peroxidase (GPx) [45]. In some reperfusion treatment studies, an increase of those enzymes [46–48] could be observed, suggesting protection of the antioxidant defense system against reactive species, while other studies reported a decrease of those enzymes [49–51]. Since these molecular changes are predominantly depending upon an increase in iNOS and subsequent oxidative stress, it is possible that oxidative stress occurs before reperfusion treatment in the onset of MI. The findings in our study, of an increase in iNOS expression during MI, allow for that hypothesis.

Whether increased activation of iNOS is beneficial or detrimental seems to depend on the occurring molecular processes in the heart. Poyton and Hendrickson [22] stated that the NO production by the iNOS l-arginine pathway is less or even not effective in hypoxia. Since iNOS mRNA expression is increased in MI hearts, iNOS may rather produce ROS than NO, due to ischemia leading to cell damages. Marfella et al. [12] evaluated the gene expression of HIF-1α, VEGF, and iNOS in myocardial biopsies of patients with and without type 2 diabetes who underwent bypass surgery. They found significantly increased iNOS mRNA and significantly higher levels of superoxide anion and nitrotyrosine in the ischemic heart in comparison with non-diabetic patients. They concluded that diabetes amplifies oxidative stress in concordance with increased iNOS expression, thereby interfering with angiogenic processes. Additionally, they detected decreased expression of HIF-1α and VEGF in these patients. In another study, Marfella et al. [14] stated that through the production of NO, iNOS provides cardioprotective effects during reperfusion. However, in a diabetic murine model, beneficial effects were not observed. The increased NO and nitrotyrosine levels produced by iNOS in those diabetic mice were found to be associated with increased myocardial injury after MI [14].

The present study showed a stronger correlation between HIF-1α and iNOS in the affected myocardium of MI hearts than between HIF-1α and VEGF, suggesting that expression patterns shift, causing HIF-1α to express iNOS instead of VEGF in the affected myocardium. No correlation between HIF-1α and iNOS was found in the non-affected myocardium, while there was a moderate monotone, but not significant correlation between HIF-1α and VEGF. Therefore, activation of iNOS transcription by HIF-1 seems to be a pathological effect, while the correlation between HIF-1α and VEGF may be physiological. However, further studies are needed, since correlation analyses can be influenced by extreme values. As expected, the monotone relations between HIF-1α and iNOS and HIF-1α and VEGF gene expression in the control hearts were significant, since HIF-1 acts as a transcription factor of iNOS and VEGF and all genes had a low expression in the control samples.

In conclusion, the stable expression of HIF-1α indicates hypoxia in affected and non-affected regions of MI hearts. The stronger correlation between HIF-1α and iNOS in the affected regions of MI hearts probably represents a pathological process and iNOS may be an early indicator of oxidative stress in MI hearts. The increased expression of iNOS in non-affected areas of MI hearts suggests that even before total artery occlusion and onset of the inflammatory reactions, pathological processes already occur in the cardiomyocytes. The significant correlation between HIF-1α and iNOS expression supports the assumption that hypoxia and oxidative stress may trigger these early changes. Therefore, iNOS expression could be considered as an indicator of early MI in forensic/clinical pathology.

The importance of biomarkers in forensics to differentiate between different cardiac events such as sudden cardiac death, arteriosclerosis, and MI is underlined by Pinchi et al. and Dlouhá et al. [52, 53]. They stated that the pathology of MI needs to be investigated as a cause of sudden cardiovascular death and therefore tested certain microRNAs for their potential of distinguishing between sudden cardiac death and MI. Whether mRNAs such as iNOS, or microRNAs, known to suppress the iNOS mRNA expression, have the same diagnostic power remains to be evaluated in further studies. Furthermore, the development of biomarkers may be helpful for personalized, pathophysiological guided therapies in repair of the infarcted myocardium, to provide information on the intensity and profile of the immune response [54].

### Study limitations

The control group is rather small. It is not excludable that an increase in the iNOS mRNA expression in healthy hearts is possible with a growing and older control group. However, since only in one healthy sample a very slight iNOS upregulation was found and the group is very strictly defined, this seems unlikely. Furthermore, the chosen calibrator sample matched best the biologically dominant characteristics of the study group.

## Electronic supplementary material

ESM 1 (DOCX 16 kb)
