# Supplementary material for: Changes in gene expression patterns in postmortem human myocardial infarction
Source: Int J Legal Med. 2020 May 12;134(5):1753–63. doi: 10.1007/s00414-020-02311-2 (PMC7417407; doi:10.1007/s00414-020-02311-2)
Supplement: Supplementary file 1 — (DOCX 16 kb) [file 414_2020_2311_MOESM1_ESM.docx]

| **Sample/Template** | **Details** |
| --- | --- |
| Source | Infarcted/normal cardiac tissue |
| Method of preservation | Preservation in RSS at -80°C |
| Storage time | No storage |
| Handling | Fresh |
| Extraction method | TriZol |
| RNA:DNA free | Probe spans exons, no RT control, DNA digestion |
| Concentration | Qubit measurements, see M&M section |
| RNA: Integrity | TapeStation measurements, see M&M section |
| Inhibition-free | No RT controls and NTCs were used and showed no inhibition in the qPCR |
| **RT/PCR** | **Details** |
| Protocols | See M&M section |
| Reagents | See M&M section |
| Duplicate RT | Technical replicates (Triplicates) were used and an average Cq for each sample was determined |
| NTC | No Cq value was measured |
| NAC | - |
| Positive control | - |
| **Data analysis** | **Details** |
| Specialist Software | Dataassist |
| Statistical justification | n=22 biological replicates |
| Transparent, validated normalisation | Reference genes, tested by Koppelkamm et al [33] and validated by geNorm [37] |

**Table S1: “MIQE précis” checklist for authors. For further information see M&M section in manuscript**
